# The impact of the GLOSSY2 and GLOSSY2-LIKE BAHD-proteins in affecting the product profile of the maize fatty acid elongase

**DOI:** 10.3389/fpls.2024.1403779

**Published:** 2024-07-11

**Authors:** Liza Esther Alexander, Dirk Winkelman, Kenna E. Stenback, Madison Lane, Katelyn R. Campbell, Elysse Trost, Kayla Flyckt, Michael A. Schelling, Ludmila Rizhsky, Marna D. Yandeau-Nelson, Basil J. Nikolau

**Affiliations:** ^1^ Roy J. Carver Department of Biochemistry, Biophysics and Molecular Biology, Iowa State University, Ames, IA, United States; ^2^ Center for Metabolic Biology, Iowa State University, Ames, IA, United States; ^3^ Department of Genetics, Development, and Cell Biology, Iowa State University, Ames, IA, United States

**Keywords:** fatty acid elongase, very long chain fatty acids, GLOSSY2, GLOSSY2-LIKE, BAHD proteins, maize, cuticular waxes, yeast

## Abstract

The maize *glossy2* and *glossy2-like* genes are homologs, which encode proteins that belong to the BAHD family of acyltransferases. *In planta* genetic studies have demonstrated that these genes may be involved in the elongation of very long chain fatty acids (VLCFAs) that are precursors of the cuticular wax fraction of the plant cuticle. VLCFAs are synthesized by a fatty acyl-CoA elongase complex (FAE) that consists of four component enzymes. Previously, we functionally identified the maize FAE component enzymes by their ability to complement haploid *Saccharomyces cerevisiae* strains that carry lethal deletion alleles for each FAE component enzyme. In this study we used these complemented haploid strains and wild-type diploid strains to evaluate whether the co-expression of either GLOSSY2 or GLOSSY2-LIKE with individual maize FAE component enzymes affects the VLCFA product-profile of the FAE system. Wild-type diploid strains produced VLCFAs of up to 28-carbon chain length. Co-expression of GLOSSY2 or GLOSSY2-LIKE with a combination of maize 3-ketoacyl-CoA synthases stimulated the synthesis of longer VLCFAs, up to 30-carbon chain lengths. However, such results could not be recapitulated when these co-expression experiments were conducted in the yeast haploid mutant strains that lacked individual components of the endogenous FAE system. Specifically, lethal yeast mutant strains that are genetically complemented by the expression of maize FAE-component enzymes produce VLCFAs that range between 20- and 26-carbon chain lengths. However, expressing either GLOSSY2 or GLOSSY2-LIKE in these complemented strains does not enable the synthesis of longer chain VLCFAs. These results indicate that the apparent stimulatory role of GLOSSY2 or GLOSSY2-LIKE to enable the synthesis of longer chain VLCFAs in diploid yeast cells may be associated with mixing plant enzyme components with the endogenous FAE complex.

## 1 Introduction

The plant cuticle provides the organism with the first physical barrier from deleterious agents in the environment. It consists of the polyester cutin, which is embedded and coated with a complex mixture of unique, solvent-extractable lipids, commonly referred to as the cuticular waxes. These cuticular waxes are primarily a mixture of linear very long chain fatty acids (VLCFAs) and derivatives (e.g., aldehydes, alcohols, hydrocarbons, ketones, and wax esters), predominantly of 24-carbon atoms and longer (Samuels et al., 2008). Because the cuticle is produced by a single layer of epidermal cells and secreted to the plant surface, the molecular mechanisms that determine this process have been difficult to characterize by classical biochemical strategies. However, mutant alleles that affect its deposition have been a powerful research tool to deciphering the metabolic processes that underlie the deposition of cuticular waxes. These include the *glossy* mutants of maize (Bianchi et al., 1985; Schnable et al., 1994), and *eceriferum* mutants of Arabidopsis (Koornneef et al., 1989).

The maize *glossy2* (*gl2*) gene was initially identified in a mutant stock that failed to accumulate normal levels of cuticular waxes on seedling leaves (Hayes and Brewbaker, 1928). Subsequently, the molecular isolation of *gl2* (Tacke et al., 1995) led to the realization that it is a homolog of the Arabidopsis *CER2* gene (Xia et al., 1996). Both *gl2* and *cer2* mutations reduce the normal accumulation of cuticular waxes in maize and Arabidopsis, respectively, and both these mutations appear to affect the ability to elongate a specific chain-length of fatty acids. In maize, the *gl2* mutation appears to block the ability to elongate fatty acids beyond the 30-carbon chain length (Bianchi et al., 1975), whereas in Arabidopsis the *cer2* mutation affects the ability to elongate fatty acids beyond 26- or 28-carbon chain lengths (McNevin et al., 1993; Jenks et al., 1995; Negruk et al., 1996).

At the time of these initial molecular characterizations, the GL2 (Tacke et al., 1995) and CER2 (Xia et al., 1996) proteins were novel, and their biochemical functionality was unclear. However, upon subsequent parallel molecular and biochemical characterization of a larger collection of proteins (St-Pierre and Luca, 2000), it became obvious that these two proteins are members of the BAHD family of enzymes that share a characteristic active site motif, the HXXXDX-motif, which catalyze acyltransferase reactions required in the biosynthesis of a variety of specialized metabolites (D’Auria, 2006; Moghe et al., 2023). BAHD acyltransferases were apparently important during the terrestrial colonization by plants, evidenced by the evolutionary expansion of the number of BAHD genes encoded by angiosperm genomes (~50-200 genes per genome). This compares to fungal genomes, which appear to be the evolutionary origin of the BAHD protein as they contain fewer than five BAHD genes per genome (Kruse et al., 2022). This evolutionary expansion and diversification of BAHD proteins appears to have supported the establishment of a wide range of biosynthetic machinery that generates the rich array of specialized metabolites that occur in the plant kingdom.

BAHD proteins have been phylogenetically classified into eight major clades, each of which display unique abilities to catalyze the acylation of different types of substrates (D’Auria, 2006; Kruse et al., 2022; Moghe et al., 2023). The common feature among these biochemically characterized proteins is the ability to acylate either an alcohol or amine functional group, generating either ester or amide products, respectively. The exception to this generalization is the clade that contains the Arabidopsis CER2 and CER2-LIKE family of proteins (Xia et al., 1996; Haslam et al., 2015, 2017; Haslam and Kunst, 2020) and the maize GL2 and GL2-LIKE proteins (Tacke et al., 1995; Alexander et al., 2020). Although heterologous expression studies in yeast have indicated that the CER2 and CER2-LIKE proteins interact with one of the enzyme components of the fatty acid elongase (FAE) system, and thereby affect the fatty acid product profile of the system (Haslam et al., 2012; Haslam and Kunst, 2013; Haslam et al., 2015, 2017; Wang et al., 2017; Haslam and Kunst, 2020; Gonzales-Vigil et al., 2021), the exact biochemical mechanism of how this is achieved remains unclear.

The FAE complex carries out the elongation of preexisting 16- and 18-carbon fatty acyl-CoAs to acyl chains of 20 carbons and longer, using malonyl-CoA as the elongating substrate. This complex is composed of four enzymatic components that iteratively catalyze cycles of Claisen condensation-reduction-dehydration and reduction reactions, resulting in the elongation of the acyl chain by 2-carbon atoms per cycle (Leonard et al., 2004). The FAE system is metabolically significant because it generates very long chain fatty acyl-CoAs, which are used as substrates to assemble such complex lipids as membrane phospholipids, storage lipids, sphingolipids (Harrison et al., 2018; Haslam and Feussner, 2022), eicosanoids (Smith, 1989; Thulasingam and Haeggström, 2020), and the plant cuticle (Samuels et al., 2008; Yeats and Rose, 2013; Barbero, 2016).

Underlying the phylogenetic diversity of these VLCFA-derived lipids, the plant FAE system displays genetic and biochemical diversity among the enzymatic components that constitute the complex (Campbell et al., 2019). Specifically, two distinct types of enzymes catalyze the Claisen condensation reactions of FAE; these are 3-ketoacyl-CoA synthases (KCSs), initially identified as the product of the Arabidopsis *FAE1* gene (James et al., 1995), and Elongation-defective proteins (ELOs), which were initially identified in yeast (Toke and Martin, 1996; Oh et al., 1997). The KCS enzymes occur exclusively in plants, whereas ELOs are present in plants, fungi, and animals (Leonard et al., 2004). The three other component-enzymes of the FAE complex are: i) 3-ketoacyl-CoA reductase (KCR), ii) 3-hydroxyacyl-CoA dehydratase (HCD) and iii) enoyl-CoA reductase (ECR). Apart from the KCR component, which was initially identified as the product of the maize *glossy8a* gene (Xu et al., 1997, 2002), the plant homologs of HCD and ECR have been identified by the genetic complementation of yeast strains that carry null allele mutations in genes that encode these functions (Gable et al., 2004; Bach et al., 2008; Campbell et al., 2019), namely the yeast *PHS1* (Denic and Weissman, 2007) and *TSC13* (Kohlwein et al., 2001) genes, respectively.

The maize genome encompasses considerable genetic redundancy among these enzymatic components, particularly in the enzymes that catalyze the first two reactions of the FAE cycle; there are 26 genes encoding for the KCS-type enzyme, possibly up to six genes encoding the ELO-type enzyme (Campbell et al., 2019; Stenback et al., 2022), and two genes encoding the KCR enzyme (Dietrich et al., 2005). In contrast, only single copy genes of the HCD and ECR component enzymes have been characterized to date (Campbell et al., 2019). The molecular genetic characterizations of the Arabidopsis CER2 and four CER2-LIKE proteins indicate that they interact with the KCS enzymatic component of FAE to affect the chain-length products of the FAE system (Haslam and Kunst, 2013; Haslam et al., 2015, 2017; Haslam and Kunst, 2020). The current study is premised on prior experiments, which indicated that one of the maize paralogs, GL2-LIKE, is a functional homolog of CER2, whereas the other, GL2, appears not to be a functional homolog. Both GL2 and GL2-LIKE share a high degree of sequence homology to CER2, including the canonical BAHD HXXXDX-catalytic motif (Alexander et al., 2020).

This study is focused on understanding the functionality of the GL2 and GL2-LIKE proteins in the context of the functionality model described above for the Arabidopsis homologs (i.e., CER2 and CER2-LIKE proteins) (reviewed by Haslam et al., 2017). The experiments described herein take advantage of the *in vivo* yeast-based system that has previously been used to functionally identify the individual components of the maize FAE complex (Campbell et al., 2019; Stenback et al., 2022), which made it possible to mix and match individual maize FAE enzyme components with either GL2 or GL2-LIKE proteins, and thereby test whether these maize BAHD proteins modify the *in vivo* generated VLCFA product-profile of the resulting yeast strains. The results of these experiments suggest that the apparent stimulatory role of GL2 or GL2-LIKE to enable the synthesis of longer chain VLCFAs may be associated with mixing plant components with the endogenous FAE enzyme complex.

## 2 Materials and methods

### 2.1 Molecular cloning

The ORFs coding for the GL2 (GRMZM2G098239; Zm00001d002353) and GL2-LIKE (GRMZM2G315767; Zm00001d024317) proteins were codon-optimized for expression in yeast using GeneOptimizer (GeneArt, LifeTechnologies) and in Arabidopsis (OptimumGene (GenScript; www.genscript.com), respectively. These sequences were chemically synthesized (GenScript; GeneArt, LifeTechnologies). These DNA fragments were cloned into high-copy episomal yeast plasmids, pAG426 (URA3) or pAG423 (HIS3) (Invitrogen, Carlsbad, CA) (Alberti et al., 2007) using either the Gateway^®^ cloning system (Invitrogen, Carlsbad, CA) or In-Fusion^®^ cloning system (Takara Bio USA, Inc., Mountain View, CA). Depending on the experiment, expression from the episomal plasmids was under the control of a galactose-inducible promoter (*GAL1*) or the constitutive glyceraldehyde-3-P-dehydrogenase (*GPD*) promoter. All recombinant yeast shuttle vectors were confirmed by DNA sequencing and were maintained in *E. coli* TOP10 cells (Invitrogen, Carlsbad, CA), using Luria Bertani (LB) media supplemented with the appropriate antibiotics.

### 2.2 Yeast Strains and Media

The yeast strains, INVSc1, BY4743, BY4741, and BY4742 were obtained from Open Biosystems (ThermoFisher Scientific, Rockford, IL) and maintained in YPD (yeast peptone dextrose) or synthetic complete (SC) dropout media. Yeast strains carrying mutations in the endogenous FAE component genes and complemented by maize FAE component genes were previously described (Campbell et al., 2019; Stenback et al., 2022). All yeast strains expressing the maize GL2 or GL2-LIKE proteins were selected by their ability to grow on minimal medium (SD) lacking the appropriate amino acid or nucleobase (e.g., uracil). The induction of expression from the *GAL1* promoter was accomplished by replacing glucose with 2% (w/v) galactose in SC medium. Yeast cultures were grown according to standard procedures in appropriate media at 30°C (Adams and Kaiser, 1998). For fatty acid analysis experiments, all strains were grown for 72 hours with exception of the INVSc1 strain that expressed maize proteins from the *GAL1* promoter; these strains were grown for 48 hours.

### 2.3 Yeast transformation

Plasmids were transformed into yeast using a standard lithium acetate transformation protocol (Gietz and Woods, 2002). Briefly, 3 µL of salmon sperm DNA, 1 µg of plasmid DNA, and 100 µL transformation mix (200 µL of 2 M lithium acetate; 800 µL of 50% (w/v) PEG; 7.7 µL of β-mercaptoethanol) were added to a 1.5 mL microcentrifuge tube and mixed by vortexing. Yeast cells from a large colony were added to the mix, vortexed, and incubated at 37°C for 30 min. Samples were then centrifuged at 900 g for 5 min, and the cell pellet was resuspended in 200 µL of sterile water and plated on selective solid media. The plates were incubated at 30°C for 2-3 days. The non-recombinant vector was transformed into each strain and used as a control.

### 2.4 Fatty acid analysis

Cell pellets were lyophilized, and after the addition of 10 μg of nonadecanoic acid as an internal standard, fatty acids were extracted and converted to methyl esters by the addition of 1 mL 5% sulfuric acid in methanol, followed by incubation of the mixture at 80°C for 1 h. After cooling, 2 mL of 0.9% (w/v) aqueous NaCl was added, and fatty acid methyl esters were extracted thrice with 1 mL of 4:1 hexane: chloroform. After each extraction, phases were separated and the organic phase was removed and pooled, and samples were dried under a stream of nitrogen gas to a final volume of ~250 µL. One microliter of each sample was analyzed by gas chromatography-flame ionization detection (GC-FID) or gas chromatography-mass spectrometry (GC-MS).

GC-FID analysis was conducted with an Agilent 6890 GC, equipped with a DB-1 MS capillary column (15 m x 0.25 mm x 0.25 μm, Agilent 122-0112). Chromatography was conducted with helium gas, at a flow-rate of 1.2 mL/min, and an inlet temperature at 280 °C. The column oven temperature was initially held at 80°C for 1 min, then ramped at 15°C/min to 230°C and held for 2 min, and then ramped at 15°C/min to 340°C and held there for 2 min. For peak identification purposes, chromatograms were compared with fatty acid methyl ester standards (8:0-30:0) and parallel GC-MS analyses were queried against the NIST 14 Mass Spectral Library. Samples that were analyzed by GC-MS were first silylated with 50 µL *N*,*O*-Bis(trimethylsilyl)trifluoroacetamide (BSTFA) with 1% trimethylchlorosilane (TMCS) (Sigma-Aldrich), prior to chromatography.

### 2.5 Statistical analysis

Statistical significance of the differences in fatty acid profiles were determined by analyzing 3-6 replicates of each yeast strain, as defined in the captions of each figure. In all cases, statistical comparisons are made only from cultures that were grown in parallel in the same incubator. Fatty acid abundance data are reported in **Supplementary Table 1**, and statistical significance between genotypes was determined by Tukey’s Honest Significant Difference (HSD) test following ANOVA. The p-values for all Tukey’s HSD tests for each experiment are included in **Supplementary Table 2**.

### 2.6 Western blot analysis

Protein extracts were prepared from yeast cell pellets using the YeastBuster Protein Extraction Reagent, (Novagen Sigma-Aldrich, Inc., St. Louis, MO). In brief, weighed cell pellets were suspended in YeastBuster Reagent (5 ml/g cell pellet) and 0.5 M Tris(hydroxypropyl) phosphine (50 µl/g cell pellet). The cell suspensions were agitated for 15-20 min at room temperature and then centrifuged at 16,000 *g* for 20 min at 4°C. The supernatant was collected and subjected to SDS-PAGE, and proteins were electrophoretically transferred from the polyacrylamide gel to a nitrocellulose membrane using a Bio-Rad Criterion™ blotter (Bio-Rad, Hercules, CA). Membranes were first incubated in a solution of 5% (w/v) non-fat milk powder, dissolved in TBST buffer (0.1% (v/v) Tween 20, 0.15 M NaCl, 2 mM KCl, 20 mM Tris-HCl, pH 7.5) that contained GL2-specific antiserum (1:1000 dilution) (Alexander et al., 2020), and then with a solution containing horseradish peroxidase-linked to goat anti-mouse IgG antibody (Bio-Rad) (1:1000 dilution). After extensive washing with TBST buffer, the antigen-antibody complexes were detected using the Pierce ECL chemi-luminescent detection system (ThermoFischer Scientific) and visualized on the ChemiDoc XRS+ gel documentation system (Bio-Rad).

### 2.7 Reverse transcription-PCR analysis

Yeast strains expressing maize proteins were grown in liquid cultures and cells were pelleted by centrifugation. RNA was extracted from the resultant cell pellets utilizing the RNeasy Mini Kit (Qiagen, Germantown, MD) according to manufacturer’s instructions. Contaminating DNA was removed from the RNA samples by treatment with RQ1 RNase-Free DNase (Promega, Madison, WI), and 1 µg of RNA was used for cDNA synthesis using either the Superscript IV Synthesis Kit (ThermoFisher Scientific) or PrimeScript Synthesis Kit (Takara Bio USA, San Jose, CA) according to manufacturer’s instructions. PCR was conducted using the resultant cDNA as template, GoTaq Green Master Mix (Promega) and gene-specific primer pairs to assess the expression of *ZmKCS6* (Primer 1: 5’-GTGAACCTCAAGCACGTCAA-3’ and Primer 2: 5’-CTCTTGTCGTCGTCGCTGAT-3’), *GL2* (Primer 1: 5’-ATGGTTTTCGAACAACACGAAG-3’ and Primer 2: 5’-TTAAGCAACATGTAAAGCAGAACCC-3’), and *GL2-LIKE* (Primer 1: 5’-ATGGTTGTTGAGGCTAACTCTG-3’ and Primer 2: 5’-TCAAGCAACTCTAAGTGCATCC-3’). *ZmKCS5* expression was assessed by utilizing Q5 High-Fidelity Polymerase with GC enhancer (NEB, UK) with gene-specific primer pairs (Primer 1: 5’- CAGAAGAACCTGCAGCTGTC-3’ and Primer 2: 5’- GCCGCTGCCGAAGCCGATCTGCCAG-3’). Each cDNA sample was also subjected to PCR analysis by using primers specific for the yeast *GLC7* gene (Primer 1: 5’-CCAGATCTATATTCATAAAGCAACCC-3’ and Primer 2: 5’-GATAATTAGATTCTGGCGGGAATC-3’). This test PCR assay established that all cDNA samples lacked the *GLC7* intron, indicating that the template samples were not contaminated by genomic DNA. The thermal cycling program for PCR was initiated at 95°C for 3 min, and then 35 cycles of incubations at 95°C for 30 s, 56°C for 45 s, and 72°C for 1.5 min; followed by a final extension step at 72°C for 5 min.

### 2.8 Accession numbers

Sequence data from this article can be found in the GenBank/EMBL data libraries under accession numbers: GRMZM2G098239 and Zm00001d002353 (*Gl2*); GRMZM2G315767 and Zm00001d024317 (*Gl2-like*); GRMZM2G393897 and Zm00001d009608 (ZmKCS4); AC233893.1_FG003 and Zm00001d048061 (ZmKCS5); GRMZM2G164974 and Zm00001d028241(ZmKCS6); GRMZM2G160417 and Zm00001d039053 (ZmKCS15); AC205703.4_FG006 and Zm00001d017111(*Gl8a*; ZmKCR1); GRMZM2G087323 and Zm00001d050992 (*Gl8b*; ZmKCR2); GRMZM2G151087 and Zm00001d039856 (ZmHCD).

## 3 Results

### 3.1 Effect of co-expressing maize GL2 or GL2-LIKE with maize KCS homologs in wild-type diploid yeast strains

Based on the carbon chain lengths of the alkyl derivatives that occur in the cuticular waxes of maize (Bianchi et al., 1985), the maize FAE system should have the ability to produce VLCFAs as long as 34-carbon atoms. Moreover, *in planta* characterizations of mutations in the maize *gl2* gene indicate that it may be involved in the ability of plant cells to produce VLCFAs beyond 26:0 and 28:0, and up to 34:0 (Bianchi et al., 1975; Alexander et al., 2020). Therefore, analogous to the Arabidopsis FAE system, where interactions between the Arabidopsis CER2-LIKE proteins and KCS paralogs stimulate the terminal elongation cycle(s) of the FAE system to generate longer chain VLCFAs (Haslam et al., 2017), yeast expression experiments evaluated whether GL2 or GL2-LIKE could affect the VLCFA profiles produced by co-expression with ZmKCS homologs. Initial experiments were conducted with ZmKCS5 or ZmKCS6, which are the closest homologs to the Arabidopsis *CER6*-encoded KCS (Campbell et al., 2019) that specifically interact with CER2 in the Arabidopsis FAE system (Haslam et al., 2015, 2017).

**Figure 1:**
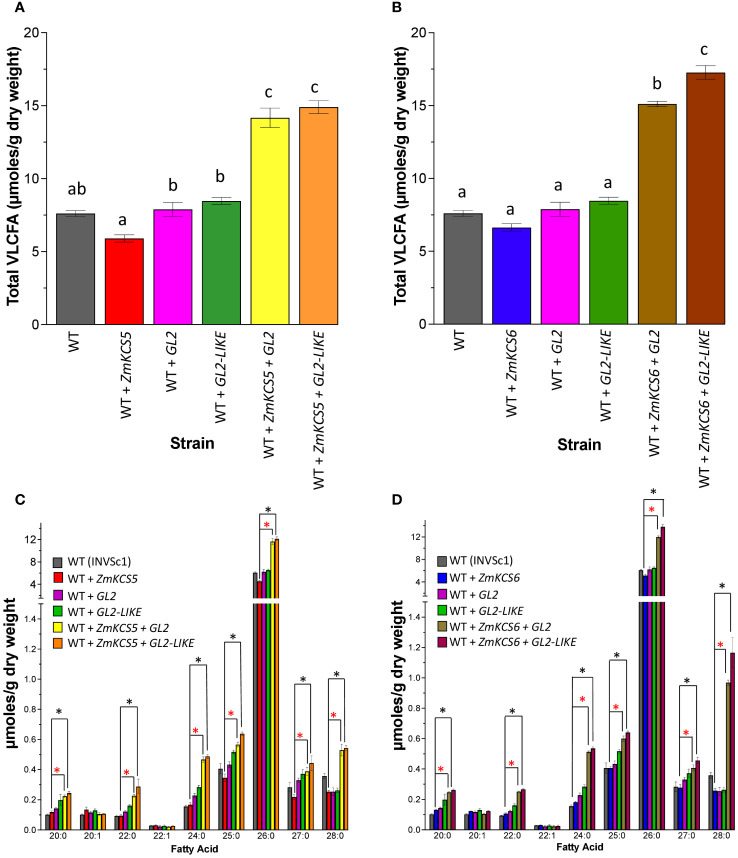
VLCFA accumulation in the INVSc1 yeast diploid strain co-expressing GL2 or GL2-LIKE with ZmKCS isozymes. **(A)** VLCFA accumulation in WT (INVSc1) and the isogenic yeast strains expressing individually or in combination ZmKCS5 and GL2 or GL2-LIKE. **(B)** VLCFA accumulation in WT (INVSc1) and the isogenic yeast strains expressing individually or in combination ZmKCS6 and GL2 or GL2-LIKE. **(C)** VLCFA composition in WT (INVSc1) and the isogenic yeast strains expressing individually or in combination ZmKCS5 and GL2 or GL2-LIKE. **(D)** VLCFA composition in WT (INVSc1) and the isogenic yeast strains expressing individually or in combination ZmKCS6 and GL2 or GL2-LIKE. All maize genes were episomally expressed under the transcriptional control of the galactose-inducible *GAL1* promoter. All data are the average ± standard error (n = 6). Statistical significance of differences among all yeast strains was determined by Tukey’s HSD test (**Supplementary Table 2**). In **(A, B)**, different letters above each data bar indicate statistically significant differences among the strains (p-value ≤ 0.05). In **(C, D)**, asterisks identify statistically significant differences (p-value ≤ 0.05) between the strain expressing only a ZmKCS isozyme and the strain co-expressing the ZmKCS isozyme with GL2 (red asterisk) or with GL2-LIKE (black asterisk).

**Figure 1** shows the results of such experiments using the diploid INVSc1 strain as the expression host. The wild-type INVSc1 strain yields a VLCFA titer of ~7.5 µmol/g dry weight, and these VLCFAs range from 20:0 to 28:0, with the most abundant VLCFA being 26:0. The individual expression of ZmKCS5, ZmKCS6, GL2 or GL2-LIKE does not significantly affect the VLCFA titers, as compared to the wild-type host strain (**Figures 1A, B**). However, the co-expression of ZmKCS5 (**Figures 1A, C**) or ZmKCS6 (**Figures 1B, D**) with either GL2 or GL2-LIKE induced an approximately 2-fold increase in the VLCFA titer (**Figures 1A, B**), and this is particularly associated with increased accumulation of the 24:0, 26:0 and 28:0 VLCFAs (**Figures 1C, D**). The fact that these increased titers occur only upon the co-expression of ZmKCS and GL2 homologs, and not when these maize proteins were individually expressed, is indicative that the GL2 homologs affect the activity of the ZmKCS proteins; possibly via interactions between the co-expressed proteins, analogous to the CER2-KCS interactions in Arabidopsis (Haslam et al., 2017).

However, these results do not recapitulate the *in planta* expectation based on the *gl2* mutant phenotype, which indicates that this gene may be involved in the ability of plant cells to produce VLCFAs of beyond 28:0 and up to 34:0 (Bianchi et al., 1975; Alexander et al., 2020). Therefore, we considered whether this may be associated with either the promoter that was used in the co-expression experiments (i.e., the galactose inducible *GAL1* promoter) or by the particular yeast strain that was used, INVSc1, whose provenance is proprietary, and unknown. Hence, both the GL2 and GL2-LIKE proteins were individually co-expressed with either ZmKCS5 or ZmKCS6 under the control of the constitutive, glyceraldehyde-3-phosphate dehydrogenase (*GPD*) promoter (Bitter et al., 1987) in the diploid strain, BY4743, which has a well-defined pedigree (Brachmann et al., 1998).

**Figure 2:**
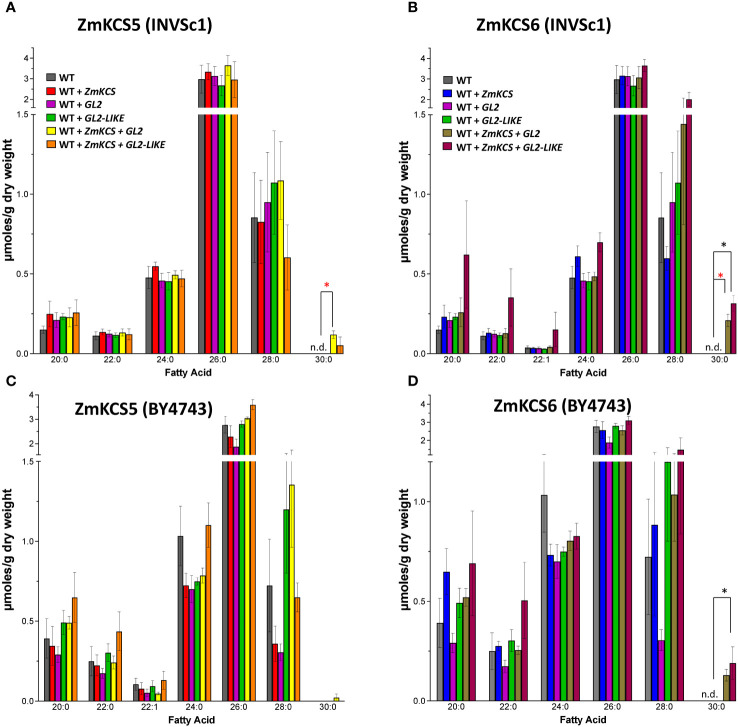
VLCFA accumulation in the yeast diploid strains, INVSc1 and BY4743, co-expressing GL2 or GL2-LIKE with ZmKCS isozymes.**(A)** VLCFA composition in WT (INVSc1), and the isogenic yeast strains expressing individually or in combination ZmKCS5 and GL2 or GL2-LIKE. **(B)** VLCFA composition in WT (INVSc1), and the isogenic yeast strains expressing individually or in combination ZmKCS6 and GL2 or GL2-LIKE. **(C)** VLCFA composition in WT (BY4743) and the isogenic yeast strains expressing individually or in combination ZmKCS5 and GL2 or GL2-LIKE. **(D)** VLCFA composition in WT (BY4743) and the isogenic yeast strains expressing individually or in combination ZmKCS6 and GL2 or GL2-LIKE. All maize genes were episomally expressed under the transcriptional control of the constitutive *GPD* promoter. All data are the average ± standard error (n = 3). Statistical significance of differences among all yeast strains was determined by Tukey’s HSD test (**Supplementary Table 2**). Asterisks identify statistically significant differences (p-value ≤ 0.05) between the strain expressing only a ZmKCS isozyme and the strain co-expressing the ZmKCS isozyme with GL2 (red asterisk) or with GL2-LIKE (black asterisk).

In contrast to the results obtained with the *GAL1* promoter, these experiments with the *GPD* promoter in either the INVSc1 or BY4743 diploid strains generated qualitatively different VLCFA profiles (**Figure 2**) that more accurately recapitulate the *in planta* chemotypes expected for the GL2 protein (Bianchi et al., 1975). Specifically, in both the INVSc1 (**Figures 2A, B**) and BY4743 (**Figures 2C, D**) strains, the co-expression of GL2 or GL2-LIKE with either ZmKCS5 or ZmKCS6 induced the accumulation of 30:0 VLCFA, a product that was undetectable in the other strains that singularly expressed ZmKCS5, ZmKCS6, GL2 or GL2-LIKE (compare **Figures 1**, **2**). These distinct observations between the use of the *GPD* and *GAL1* promoters in different diploid strains indicate that additional insights are required to dissect the complexity of co-expressing FAE components in heterologous biological systems, such as plant FAE components in yeast strains that also carry an endogenous FAE system.

### 3.2 Effect of co-expressing maize GL2 or GL2-LIKE with maize KCS isozymes as replacements of the yeast ELO3 component-enzyme of FAE

**Figure 3:**
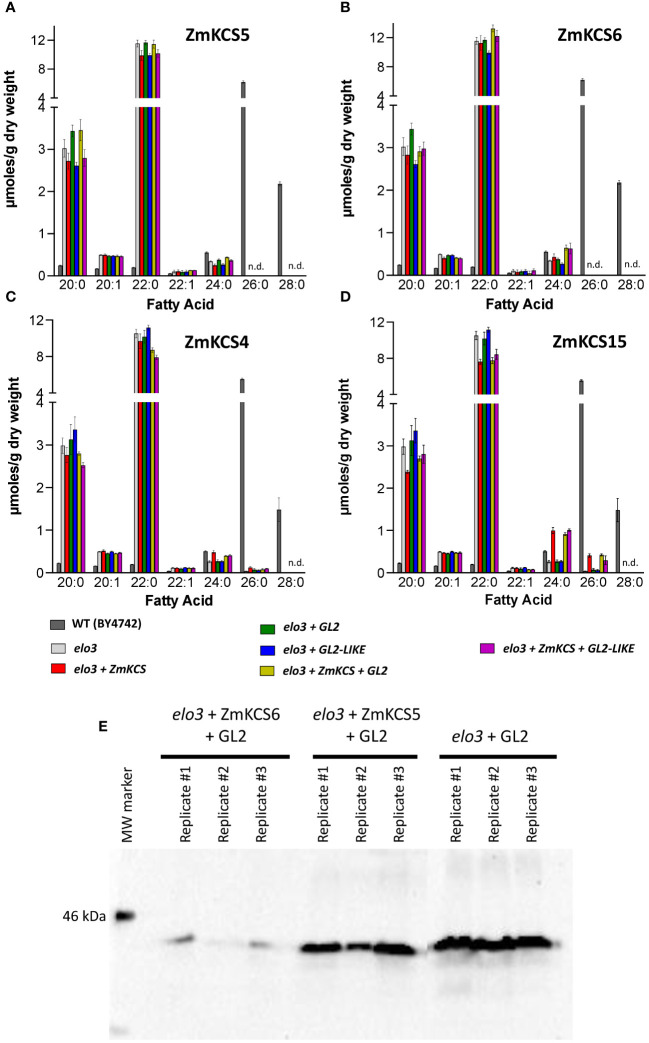
VLCFA accumulation in the yeast haploid strain (BY4742) carrying an *elo3* null allele co-expressing GL2 or GL2-LIKE with ZmKCS isozymes. **(A)** VLCFA composition of yeast *elo3* mutant strains expressing individually or in combination ZmKCS5 and GL2 or GL2-LIKE. **(B)** VLCFA composition of yeast *elo3* mutant strains expressing individually or in combination ZmKCS6 and GL2 or GL2-LIKE. **(C)** VLCFA composition of yeast *elo3* mutant strains expressing individually or in combination ZmKCS4 and GL2 or GL2-LIKE. **(D)** VLCFA composition of yeast *elo3* mutant strains expressing individually or in combination ZmKCS15 and GL2 or GL2-LIKE. **(E)** GL2 protein expression demonstrated by Western blot analyses of the indicated strains (n=3). All maize genes were episomally expressed under the transcriptional control of the constitutive *GPD* promoter. All fatty acid composition data are the average ± standard error (n = 4). Statistical significance of differences among all yeast strains was determined by Tukey’s HSD test (**Supplementary Table 2**). There were no statistically significant differences (p-value ≤ 0.05) in VLCFA profiles between the strain expressing only a ZmKCS isozyme and the strain co-expressing the ZmKCS isozyme with GL2 or with GL2-LIKE.

To overcome the complexity of genetically adding heterologous maize FAE components to an intact FAE system (i.e., the yeast FAE), we co-expressed ZmKCS paralogs with either GL2 or GL2-LIKE in a yeast strain that lacked the analogous endogenous yeast FAE component enzyme. These experiments were conducted in a haploid strain (i.e., BY4742, which is one of the haploid progenitors of the diploid strain, BY4743 (Brachmann et al., 1998)) that carries mutations in genes coding for yeast FAE component genes. The yeast FAE system does not utilize KCS-type condensing enzymes, but rather utilizes ELO-type condensing enzymes, encoded by the *ELO1*, *ELO2* or *ELO3* genes (Toke and Martin, 1996; Oh et al., 1997). Because ELO3 has the ability to catalyze the terminal cycles of fatty acid elongation using preexisting 24:0 and 26:0 acyl-CoAs (Oh et al., 1997; Denic and Weissman, 2007), we first recapitulated the above experiments in the BY4742 haploid strain that carried an *elo3* null allele (note that this strain carries functional *ELO1* and *ELO2* genes). In these experiments we individually expressed ZmKCS4, ZmKCS5, ZmKCS6 and ZmKCS15 in the *elo3* mutant yeast strain, and compared the VLCFA profiles when these ZmKCS isozymes were co-expressed with either GL2 or GL2-LIKE (**Figure 3**). The rationale for including ZmKCS4 and ZmKCS15 isozymes is based on the fact that these two plant isozymes have been shown to be functional in yeast (Stenback et al., 2022).

As with the diploid strains, the wild-type BY4742 haploid strain produced VLCFAs that range between 20 and 28 carbon chain lengths, with 26:0 accounting for 80% of the VLCFAs. The effect of the *elo3* mutation was to eliminate the accumulation of 26:0 and 28:0, and thus the predominant VLCFA in this mutant yeast strain was 24:0. The expression of ZmKCS5 or ZmKCS6 in the *elo3* mutant strain had no effect on the fatty acid profiles (**Figures 3A, B**). However, the expression of ZmKCS4 or ZmKCS15 in the *elo3* mutant strain resulted in partially restoring the ability of the strain to produce the longer VLCFAs; specifically these strains produced small but statistically significant quantities of 26:0 (**Figures 3C, D**). Thus, in contrast to ZmKCS4 or ZmKCS15, neither ZmKCS5 or ZmKCS6 were capable of replacing the *elo3* function. Moreover, the co-expression of either GL2 or GL2-LIKE with each of these four ZmKCS isozymes was incapable of modifying the abilities of the resulting strains to produce larger quantities of the longer chain VLCFAs (i.e., 26:0, 28:0, or 30:0) (**Figure 3**).

### 3.3 Effect of co-expressing maize GL2 or GL2-LIKE with maize KCS isozymes that functionally replace the yeast ELO function

As demonstrated by the synthetic lethality associated with the *elo2 elo3* double mutant, the ability to produce VLCFAs is essential for yeast viability (Toke and Martin, 1996; Oh et al., 1997). Moreover, prior characterizations have established that five ZmKCS isozymes (i.e., ZmKCS2, ZmKCS4, ZmKCS11, ZmKCS15, and ZmKCS20) are capable of genetically rescuing this lethality of the *elo2 elo3* double mutant (Stenback et al., 2022). Therefore, we used these rescued strains to evaluate whether the co-expression of GL2 or GL2-LIKE could modify the product profile of the FAE system, indicative of interactions with the ZmKCS isozymes.

**Figure 4:**
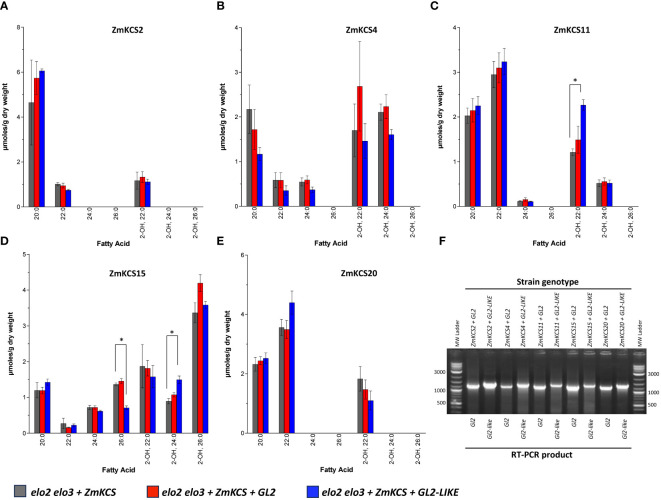
VLCFA accumulation upon co-expressing GL2 or GL2-LIKE with ZmKCS isozymes that genetically complement the lethality of the *elo2 elo3* double mutant yeast strain. VLCFA composition of yeast *elo2 elo3* double mutant strains expressing individually or in combination GL2 or GL2-LIKE and ZmKCS2 **(A)**, ZmKCS4, **(B)** ZmKCS11 **(C)**, ZmKCS15 **(D)**, and ZmKCS20 **(E)**. **(F)** RT-PCR analysis of the expression of the *Gl2* (1300-bp product) and *Gl2-like* (1400-bp product) mRNAs in the indicated yeast strains. All maize genes were episomally expressed under the transcriptional control of the *GPD* constitutive promoter. Numerical data in panels **(A–E)** are the average ± standard error (n=3). Statistical significance of differences among all yeast strains was determined by Tukey’s HSD test (**Supplementary Table 2**). Asterisks identify statistically significant differences (p-value ≤ 0.05) between the strain expressing only a ZmKCS isozyme and the strain co-expressing that ZmKCS isozyme with GL2 (red asterisk) or with GL2-LIKE (black asterisk).

As previously reported (Stenback et al., 2022), in the absence of either GL2 or GL2-LIKE, the expression of ZmKCS2 enables the elongation of fatty acids to 22-carbon chain length, whereas ZmKCS4, ZmKCS11, and ZmKCS20 enables the elongation of fatty acids to 24-carbon chain length, and ZmKCS15 can elongate fatty acids up to 26-carbon atoms (**Figure 4**). These acyl-products include VLCFAs and 2-hydroxy-VLCFAs, which are hydroxylated post-synthesis in the assembly of ceramide-based lipids (Erdbrügger and Fröhlich, 2021). The co-expression of either GL2 or GL2-LIKE with each of these ZmKCS isozymes did not cause any qualitative change in the VLCFA profiles; namely there were no new VLCFA products generated that were absent from the strains that only expressed the ZmKCS isozyme. Rather, there were statistically significant quantitative changes in the accumulation of some of the VLCFA products. For example, GL2-LIKE caused increased accumulation of 2-hydroxy-22:0 or 2-hydroxy-24:0 when it was co-expressed with ZmKCS11 (**Figure 4C**) or ZmKCS15 (**Figure 4D**), respectively; the latter change was accompanied by the decreased accumulation of 26:0 (**Figure 4D**).

### 3.4 Effect of co-expressing maize GL2 or GL2-LIKE in combination with pairs of maize KCS isozymes

**Figure 5:**
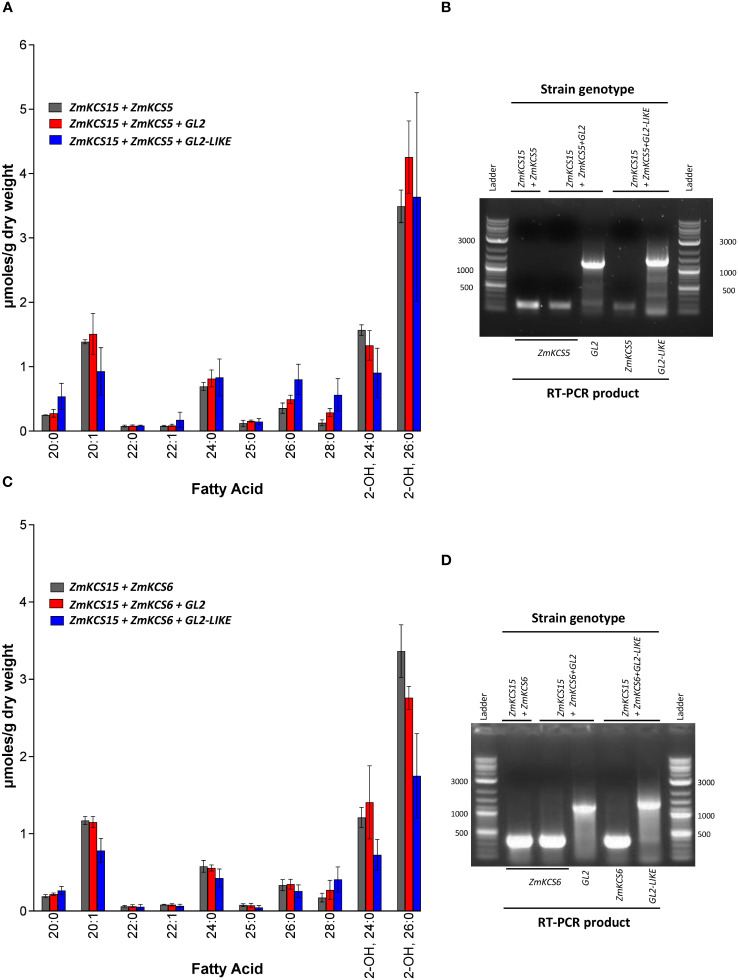
VLCFA accumulation upon co-expressing GL2 or GL2-LIKE with different combinations of ZmKCS isozymes that rescue the lethality of the *elo2 elo3* double mutant yeast strain. **(A)** VLCFA composition of yeast *elo2 elo3* double mutant strains genetically complemented by the combined co-expression of the ZmKCS15 and ZmKCS5 isozymes, in the absence or presence of either GL2 or GL2-LIKE. **(B)** RT-PCR analysis of the expression of the *Gl2* (1300-bp product), *Gl2-like* (1400-bp product) and *ZmKCS5* (168-bp product) mRNAs in the indicated yeast strains. **(C)** VLCFA composition of yeast *elo2 elo3* double mutant strains genetically complemented by the combined co-expression of the ZmKCS15 and ZmKCS6 isozymes, in the absence or presence of either GL2 or GL2-LIKE. **(D)** RT-PCR analysis of the expression of the *Gl2* (1300-bp product), *Gl2-like* (1400-bp product) and *ZmKCS6* (330-bp product) mRNAs in the indicated yeast strains. All maize genes were episomally expressed under the transcriptional control of the *GPD* constitutive promoter. Numerical data in panels **(A, C)** are the average ± standard error (n=3). Statistical significance of differences among all yeast strains was determined by Tukey’s HSD test (**Supplementary Table 2**). There were no statistically significant differences (p-value ≤ 0.05) in VLCFA profiles between the strain co-expressing combinations of ZmKCS isozymes in the absence or presence of either GL2 or GL2-LIKE.

The findings observed with the GL2 and GL2-LIKE proteins (**Figure 4**) are not consistent with the model developed with the Arabidopsis homologs of these proteins (i.e., the CER2 and CER2-LIKE proteins) (Haslam et al., 2017). This difference between the maize and Arabidopsis homologs may be associated with the fact that the CER2 and CER2-LIKE proteins mediate this effect by specifically interacting with the *CER6*- or *CER60*-encoded KCS isozymes (Haslam et al., 2015). The difference between the two systems (i.e., maize versus Arabidopsis) may be due to the fact that the ZmKCS isozymes used in the current study are in a phylogenetic clade that is distinct from the Arabidopsis *CER6*- or *CER60*-encoding KCS isozymes. In maize, the phylogenetic homologs to the *CER6*-and *CER60-*encoding KCSs are ZmKCS5 and ZmKCS6 (Campbell et al., 2019). However, because ZmKCS5 and ZmKCS6 are incapable of complementing the yeast *elo2 elo3* double mutant strain (Stenback et al., 2022), it was not possible to co-express them individually with GL2 or GL2-LIKE in the yeast system lacking the native condensing enzymes. Therefore, we co-expressed either ZmKCS5 or ZmKCS6 in the yeast *elo2 elo3* double mutant strain that was rescued by genetic complementation by the expression of ZmKCS15. This complemented strain was chosen because it was capable of producing the longest VLCFAs (i.e., up to 26:0) (Stenback et al., 2022). **Figure 5** shows that the co-expression of GL2 or GL2-LIKE with either ZmKCS5 or ZmKCS6 in the ZmKCS15-rescued *elo2 elo3* double mutant had no effect on the VLCFA profiles that were generated.

Comparing the VLCFA profiles in **Figures 4D** and **5A**, we found that the co-expression of ZmKCS15 with ZmKCS5 in the *elo2 elo3* double mutant is no different than solely expressing ZmKCS15 in this strain, and the co-expression of GL2 or GL2*-*LIKE with both ZmKCS15 and ZmKCS5 had little effect on the profile (**Figure 5A**). Similar results were obtained when ZmKCS15 expression was combined with ZmKCS6 expression; the most pronounced apparent effect, which is not statistically significant, was a 50% reduction in the accumulation of 2-hydroxy-26:0 upon the co-expression with GL2-LIKE (**Figure 5B**).

### 3.5 Effect of co-expressing GL2 and GL2-LIKE with other FAE components

Prior studies have indicated that protein-protein interactions among the FAE component enzymes are important for VLCFA biosynthesis; in the Arabidopsis system this includes interactions between CER2-LIKE proteins and the KCR, HCD and ECR components (Kim et al., 2022). Therefore, using the yeast strains that we previously developed to functionally characterize these additional components of the maize FAE system (Campbell et al., 2019), we evaluated whether the GL2 or GL2-LIKE proteins can interact with additional FAE components (i.e., ZmKCR1, ZmKCR2 and ZmHCD) and thereby affect the VLCFA product-profile of the FAE system.

**Figure 6:**
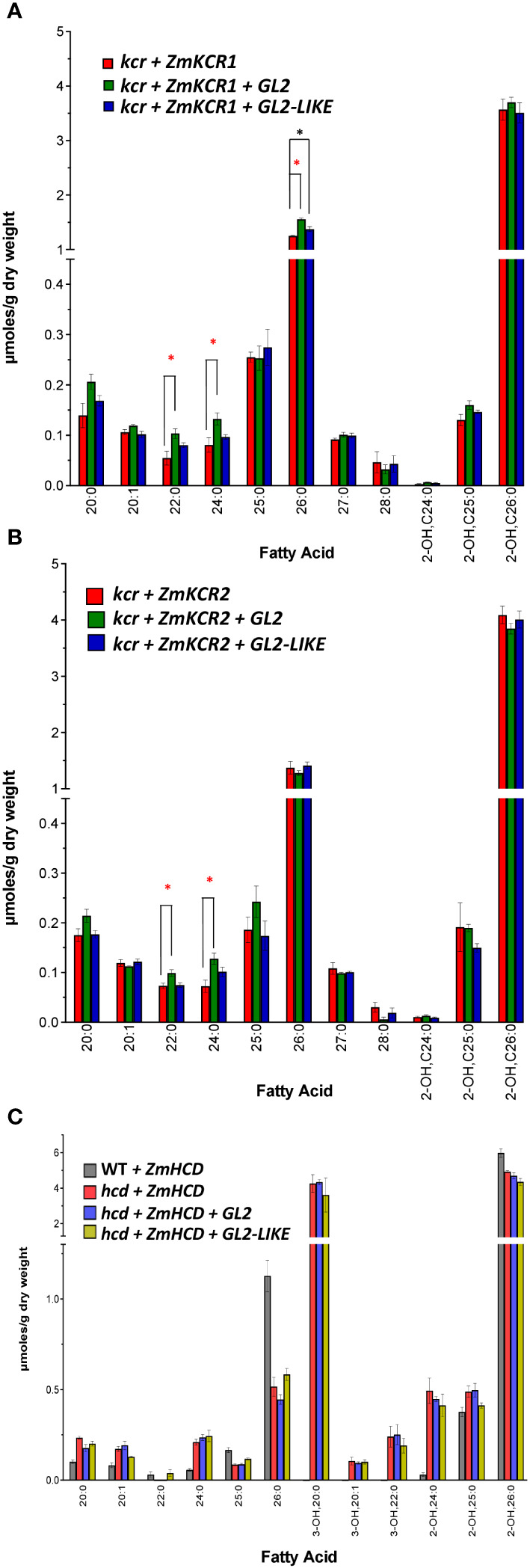
VLCFA accumulation upon co-expressing GL2 or GL2-LIKE with ZmKCR1, ZmKCR2 or ZmHCD that rescue the lethality of the yeast *kcr* or *hcd* null alleles, respectively. **(A)** VLCFA composition of the yeast *kcr* mutant strain genetically rescued by the expression of ZmKCR1, in the absence or presence of either GL2 or GL2-LIKE. **(B)** VLCFA composition of the yeast *kcr* mutant strain genetically rescued by the expression of ZmKCR2, in the absence or presence of either GL2 or GL2-LIKE. **(C)** VLCFA composition of the yeast *hcd* mutant strain genetically rescued by the expression of ZmHCD, in the absence or presence of either GL2 or GL2-LIKE. All maize genes were episomally expressed under the transcriptional control of the galactose-inducible *GAL1* inducible promoter. Data are the average ± standard error (n=4). Statistical significance of differences among all yeast strains was determined by Tukey’s HSD test (**Supplementary Table 2**). Asterisks identify statistically significant differences (p-value ≤ 0.05) between the strain expressing GL2 (red asterisk) or with GL2-LIKE (black asterisk), relative to their absence.

We evaluated if either GL2 or GL2-LIKE could affect changes in VLCFA profiles upon co-expression with either ZmKCR1 (g*l8a*) or ZmKCR2 (*gl8b*) (Xu et al., 1997, 2002; Dietrich et al., 2005). These co-expression experiments were conducted in yeast strains that lacked the endogenous yeast KCR (i.e., the *ybr159Δ* mutant strain) (Han et al., 2002). The yeast *ybr159Δ* mutation is lethal, but can be rescued by the expression of either ZmKCR1 or ZmKCR2 (Campbell et al., 2019). The resulting rescued yeast strains produced VLCFA profiles that are similar to those that occur in the wild-type strain, with 26:0 being the most abundant VLCFA (**Figure 6**). The co-expression of either GL2 or GL2-LIKE with the two maize KCR isozymes, ZmKCR1 (**Figure 6A**) or ZmKCR2 (**Figure 6B**), produced minor, but statistically significant quantitative changes in the VLCFA profiles, affecting only 22:0, 24:0, and 26:0.

Finally, using the same strategy we investigated if GL2 or GL2-LIKE interacts with ZmHCD to affect VLCFA profiles, by co-expressing the two combinations in the yeast *hcd* mutant strain (Campbell et al., 2019). **Figure 6C** compares the VLCFA profiles of the ZmHCD-rescued *hcd* mutant yeast strains, with and without the co-expression of either GL2 or GL2-LIKE. Although the VLCFA profile of the yeast strain is not altered when ZmHCD is expressed in the wild-type background, the profile is strikingly different when ZmHCD is expressed in the *hcd* mutant background. Specifically, ZmHCD complementation induces the appearance of 3-hydroxy-VLCFAs of 20 and 22 carbon chain lengths and a 50% reduction in 26:0. We suggest that this is because the maize HCD is not as efficient in replacing the endogenous yeast HCD in converting 3-hydroxyacyl-CoA to the enoyl-CoA, and thus 3-hydroxy-VLCFAs accumulate. Even so, the co-expression of ZmHCD with either GL2 or GL2-LIKE did not further alter the VLCFA profiles of the rescued strains (**Figure 6C**).

### 3.6 Molecular confirmation of the expression of maize genes in yeast

The expression of ZmKCS2, ZmKCS4, ZmKCS11, ZmKCS15 and ZmKCS20 proteins in yeast cells was genetically confirmed because their presence rescued the lethality associated with the yeast *elo2 elo3* double mutant strain (e.g., **Figure 4**). In addition, changes in VLCFA profiles in a number of yeast strains indicate that the GL2 and GL2-LIKE proteins were successfully expressed (e.g., **Figures 1**, **2**). However, in some strains that were engineered to express these proteins, the resultant VLCFA profiles were very similar to those generated by the host strain (e.g., **Figures 3**–**5**). Therefore, we sought molecular evidence to confirm successful expression of the GL2 and GL2-LIKE proteins, and ZmKCS isozymes. Such confirmation was obtained by either Western blot or RT-PCR analyses.

For example, **Figure 3E** shows Western blot analyses of protein extracts from a subset of the yeast *elo3-*mutant strains, which confirms that the GL2 protein was successfully expressed in these experiments, even though its expression did not generate a change in the VLCFA profile. Analogously, RT-PCR analysis confirmed the expression of the *Gl2* and *Gl2-like* mRNA in the yeast *elo2 elo3* double mutant strains that were genetically complemented by the expression of the ZmKCS2, ZmKCS4, ZmKCS11, ZmKCS15 or ZmKCS20 proteins (**Figure 4F**), even though the expression of *Gl2* and *Gl2-like* did not qualitatively change the VLCFA profiles (**Figures 4A–E**).

Similarly, we evaluated the expression of mRNAs encoding for GL2 or GL2-LIKE proteins and either ZmKCS5 (**Figure 5B**) or ZmKCS6 (**Figure 5D**), which were co-expressed with ZmKCS15 in the yeast *elo2 elo3* double mutant strain. This double mutant strain is only viable because of the expression of the ZmKCS15 protein (**Figure 4D**). Thus, in these strains ZmKCS15 was co-expressed with either ZmKCS5 or ZmKCS6, in the presence or absence of either GL2 or GL2-LIKE. RT-PCR analyses confirmed the expression of *Gl2* and *Gl2-like* mRNAs and either *ZmKCS5* (**Figure 5B**) or *ZmKCS6* (**Figure 5D**), even though these genetic modifications did not affect changes in the VLCFA profiles (**Figures 5A, C**).

## 4 Discussion

The BAHD class of acyltransferases was initially identified by the biochemical characterization of four enzymes that are involved in the biosynthesis of plant secondary metabolites (St-Pierre and Luca, 2000). Subsequently, hundreds of these enzymes have been characterized from diverse phylogenetic sources, and they have been classified into eight different sequence-based clades, which also segregate these enzymes according to the chemical nature of the substrate that is acylated (Kruse et al., 2022; Moghe et al., 2023). One of these clades (i.e., Clade 2) contains proteins (i.e., GL2 and CER2) whose acyltransferase activity is uncharacterized. These two proteins were genetically defined by mutant alleles that affect the normal accumulation of cuticular waxes (Hayes and Brewbaker, 1928; Koornneef et al., 1989). Following the molecular characterization of these genetic loci (Tacke et al., 1995; Negruk et al., 1996; Xia et al., 1996), the homologous *Gl2-like* locus (Alexander et al., 2020) and four homologous *CER2-LIKE* loci (Pascal et al., 2013; Haslam et al., 2015; Haslam and Kunst, 2020) were identified in maize and Arabidopsis, respectively. Additionally, homologs of these proteins have been characterized from a number of different plant species, including rice (Wang et al., 2017), onion (Liu et al., 2023), broccoli (Han et al., 2021), cabbage (Ji et al., 2021), and poplar (Gonzales-Vigil et al., 2021).

The functionality of these proteins has been based on a model developed from extensive characterizations of the Arabidopsis CER2 and CER2-LIKE proteins (reviewed by Haslam et al. (2017)). These characterizations indicate that the CER2 and CER2-LIKE proteins are modulators of the product-profile of the FAE system, mediated by physical interactions with the KCS component of the FAE system (Haslam and Kunst, 2020; Kim et al., 2022). Hence, in the presence of CER2 or CER2-LIKE, the FAE system can produce longer chain VLCFAs than in their absence (Haslam et al., 2012, 2015; Haslam and Kunst, 2020). This functionality model was primarily developed from the analyses of yeast strains that co-express Arabidopsis KCS isozymes with CER2 or CER2-LIKE proteins. Specifically, the singular expression of an Arabidopsis KCS isozyme in yeast produces VLCFAs of only 28-carbon chain length. However, co-expression of CER2 or CER2-LIKE proteins with Arabidopsis KCS isozymes enables the yeast strain to produce VLCFAs of longer chain lengths, up to 34-carbon atoms (Haslam et al., 2012, 2015; Haslam and Kunst, 2020). Moreover, this modification of the product profile of the FAE system by CER2 and CER2-LIKE proteins displays specificity, occurring only with the Arabidopsis KCS6 (*CER6*; AT1G68530) and KCS5 (*CER60*; AT1G25450) proteins, and does not occur with KCS1 (AT1G01120), KCS10 (*FIDDLEHEAD*; AT2G26250), KCS9 (AT2G16280) or KCS20 (AT5G43760) (Haslam et al., 2015). This model provides an explanation for the observed *in planta* change in the cuticular wax phenotypes expressed by the loss-of-function *cer2* or *cer2-like* mutants. Specifically, Arabidopsis *cer2* mutants do not accumulate cuticular VLCFAs and derivative products that are greater than 28-carbon chain length, whereas in the wild-type state these cuticular components are derived from 30- to 34-carbon VLCFAs (Negruk et al., 1996; Haslam et al., 2012, 2015; Haslam and Kunst, 2020).

We had previously characterized the functionality of the maize GL2 and GL2-LIKE proteins relative to CER2 function by *in planta* genetic complementation experiments, which indicated that the two maize homologs have overlapping and distinct functions. Specifically, the transgenic expression of either the GL2 or GL2-LIKE protein in Arabidopsis can complement the *cer2* mutation by restoring the production of VLCFAs of 26:0 and greater chain lengths. However, whereas the GL2-LIKE protein requires an intact HXXXDX catalytic-motif to fully complement the *cer2* function, GL2 protein can accomplish this complementation without an intact HXXXDX motif (Alexander et al., 2020).

In this study we explored the potential interactions between the GL2 or GL2-LIKE proteins with the maize FAE components, using yeast as the co-expression platform. Extrapolating from the functionality model developed for the homologous CER2 and CER2-LIKE proteins, we initially focused on the ability of GL2 or GL2-LIKE to affect the capability of ZmKCS isozymes to produce longer chain VLCFAs. We found that the effects of GL2 or GL2-LIKE were dependent on the yeast strain that was used and on the promoter that was used to drive the co-expression of these proteins. Specifically, when co-expression was controlled by the *GAL1* promoter, in the diploid INVSc1 yeast strain, both GL2 or GL2-LIKE affected the ability of ZmKCSs to produce larger quantities of the longer chain VLCFAs, without enabling additional elongation cycles that would produce even longer chain products (i.e., only producing VLCFAs of up to 28-carbon atoms). However, when co-expression was controlled by the constitutive *GPD* promoter, in two diploid strains (i.e., INVSc1 or BY4743), both GL2 or GL2-LIKE stimulated the ability of ZmKCSs to produce the longer chain VLCFAs (i.e., 30:0) that could not be produced in the absence of GL2 or GL2-LIKE.

Although the latter results with the *GPD* promoters are consistent with the functionality model developed with the Arabidopsis CER2 and CER2-LIKE proteins (Haslam et al., 2017), we considered the possibility that the inconsistent results obtained with the *GAL1* promoter in the INVSc1 strain may be associated with the complexity of co-expressing the ZmKCS isozymes with GL2 or GL2-LIKE in a host that has an intact endogenous FAE system. Adding to this complexity is the fact that the endogenous yeast FAE system does not utilize a KCS-type condensing enzyme, but rather utilizes a combination of three ELO-type condensing enzymes (ELO1, ELO2 and ELO3) (Toke and Martin, 1996; Oh et al., 1997). This complexity was partially overcome by taking advantage of yeast strains that we had previously developed, which were viable only because of the expression of maize FAE components (Campbell et al., 2019; Stenback et al., 2022). These strains carry null alleles in the endogenous FAE component genes (i.e., *elo2 elo3* double mutant, *kcr* and *hcd* mutants), which is a lethal condition. However, the lethality associated with these mutants was rescued by the expression of individual maize FAE components. The co-expression of GL2 or GL2-LIKE in these strains did not significantly affect the VLCFA profiles produced by the resulting strains, in particular there was no induction of the synthesis of longer chain VLCFAs (i.e., of 30-carbons or longer), as was expected based on the functionality model developed with the Arabidopsis *CER2* and *CER2-LIKE* genes (Haslam et al., 2017).

There are a number of potential explanations for these observations. For example, the GL2 and GL2-LIKE proteins may have functions that are distinct from the CER2 or CER2-LIKE proteins. However, our prior study, which demonstrated that the *in planta* expression of the two maize proteins can genetically complement the *cer2* mutation, is inconsistent with this explanation (Alexander et al., 2020). Another possibility is that the effect of the GL2 and GL2-LIKE proteins on FAE is masked by the fact that the yeast strains that we developed expressed a hybrid FAE system that mixed maize and yeast components. This is a very viable explanation because bimolecular fluorescence complementation and yeast two-hybrid assays have indicated that protein–protein interactions are important in the assembly of a functional FAE system (Kim et al., 2022). This is of particular significance because the yeast FAE system uses an ELO-type enzyme to catalyze the Claisen condensation reaction to generate the new carbon-carbon bond that enables the elongation of the substrate (Toke and Martin, 1996; Oh et al., 1997), whereas the interaction between CER2 or CER2-LIKE proteins with FAE is mediated through interactions with the KCS component (Haslam et al., 2017). Indeed, with the many recent synthetic biological reagents that have been developed for yeast, it is becoming possible to evaluate this last possibility by reconstituting the entire plant FAE system. Most recently, the effect of the CER2 and CER2-LIKE proteins have been demonstrated with yeast strains that comprehensively express the Arabidopsis FAE system (Batsale et al., 2023), although these strains are also co-expressing the endogenous yeast FAE system. Based upon our prior reconstitution experiments (Campbell et al., 2019; Stenback et al., 2022) it should be possible to develop viable yeast strains that are solely expressing a maize FAE system, which can be used to specifically evaluate the functionality of GL2 or GL2-LIKE in affecting VLCFA production.

## Data availability statement

The original contributions presented in the study are included in the article and **Supplementary Material**, further inquiries can be directed to the corresponding author/s.

## Author contributions

LEA: Data curation, Formal analysis, Investigation, Methodology, Supervision, Visualization, Writing – original draft. DW: Data curation, Formal analysis, Investigation, Methodology, Supervision, Visualization, Writing – original draft. KES: Investigation, Methodology, Writing – review & editing. KRC: Investigation, Methodology, Writing – review & editing. ET: Investigation, Methodology, Writing – review & editing. KF: Investigation, Methodology, Writing – review & editing. MAS: Investigation, Methodology, Writing – review & editing. LR: Investigation, Methodology, Writing – review & editing. MDY-N: Conceptualization, Funding acquisition, Project administration, Resources, Supervision, Writing – review & editing. BJN: Conceptualization, Funding acquisition, Project administration, Resources, Supervision, Visualization, Writing – review & editing. ML: Investigation, Visualization, Writing – original draft.
